# How to Diagnose and Treat Local Allergic Rhinitis: A Challenge for Clinicians

**DOI:** 10.3390/jcm8071062

**Published:** 2019-07-19

**Authors:** Ibon Eguiluz-Gracia, Natalia Pérez-Sánchez, Gádor Bogas, Paloma Campo, Carmen Rondón

**Affiliations:** Allergy Department, IBIMA-Hospital Regional Universitario de Malaga-ARADyAL, Málaga 29009, Spain

**Keywords:** local allergic rhinitis, nasal allergen challenge, allergen immunotherapy

## Abstract

Chronic rhinitis is a very common disease that can be divided in various phenotypes. Historically, the condition has been classified into the allergic rhinitis (AR) and non-allergic non-infectious rhinitis (NAR) forms, based on the results of the classical biomarkers of atopy: skin prick test and serum allergen-specific IgE However, this classification does not reflect the complexity of the rhinitis syndrome, as illustrated by the existence of non-atopic rhinitis patients who display a nasal reactivity to environmental allergens. This new phenotype has been termed local allergic rhinitis (LAR) and can be only recognized if an additional test such as the nasal allergen challenge (NAC) is integrated in the diagnostic algorithm for chronic rhinitis. Recent data shows that the NAC is a very safe and reliable technique ready for the clinical practice. LAR is a differentiated rhinitis phenotype which often commences during childhood and quickly progresses towards a clinical worsening and the association of comorbidities in other mucosal organs. Recent evidence supports the existence of a bronchial counterpart of LAR (local allergic asthma), which highlights the pathophysiological links between the upper and lower airways and reinforces the united airways concept. Importantly, several controlled studies have demonstrated the ability of allergen immunotherapy to control LAR symptoms while the therapy is being administered. This review emphasizes the need to implement the NAC in the clinical practice in order to facilitate the recognition of LAR patients, allowing for an early prescription of specific therapies with disease-modifying potential.

## 1. Introduction

Chronic rhinitis affects up to 30% of the general population in Western countries and imposes a significant burden to healthcare systems in terms of both direct and indirect costs [1]. Moreover, chronic rhinitis largely impairs quality of life and is associated to other inflammatory diseases, such as sinusitis, conjunctivitis, and asthma, further amplifying its impact [2]. Despite these deleterious effects, the condition has been historically trivialized and only in recent years has gained significant attention from physicians and researchers [1]. 

One simple classification divides the disorder between allergic rhinitis (AR) and non-allergic non-infectious rhinitis (often simplified as NAR) [3,4]. AR is a relatively homogenous condition defined by a nasal eosinophilic inflammation arising from the IgE-sensitization to seasonal or perennial aeroallergens [3]. On the other hand, NAR is a highly heterogeneous entity comprising disorders characterized by either immunological or neurogenic inflammation [4,5]. To discriminate these phenotypes, two biomarkers were historically available: a skin prick test (SPT) and the serum allergen-specific IgE (sIgE) [6]. The positivity of any of these biomarkers is used to identify atopic individuals in the clinical practice [7]. By definition, AR patients are positive for at least one of these two classical markers of atopy [3], whereas NAR individuals test negative for both [4]. 

Nevertheless, a significant proportion of healthy subjects also displays positivity for SPT or serum sIgE [7], demonstrating that the correlation with the pattern of nasal symptoms is crucial to interpret IgE-sensitization tests. In this regard, an additional in vivo biomarker like the nasal allergen challenge (NAC) can help identify the clinically relevant allergens in individual patients [8]. By definition, NAR subjects test negative for the NAC, whereas AR patients display positive responses for at least one aeroallergen [8]. Interestingly, a proportion of non-atopic rhinitis patients also test positive for the NAC [9]. This new rhinitis phenotype has been termed local allergic rhinitis (LAR) [10], and it does not fit into the classical AR–NAR dichotomy [1]. Similar to AR, LAR patients display an eosinophilic nasal inflammation [11], but unlike AR subjects, they test negative for SPT and serum sIgE [12]. Thus, a NAC is needed to establish the diagnosis of LAR [13,14]. 

Treatment options in rhinitis are guided by the disease phenotype [1,5]. Antihistamines and nasal steroids are able to control the symptoms of AR patients and of those NAR individuals with eosinophilic nasal inflammation [15]. On the other hand, these drugs are less effective in NAR patients with neurogenic inflammation [5]. Moreover, allergen immunotherapy (AIT) is the only existing etiological treatment for AR subjects [16], as it does not only control the symptoms, but also induces a long-lasting beneficial effect and modifies the natural course of the disease [17]. Interestingly, recent evidence suggests that AIT might have a similar beneficial effect in LAR individuals [18,19,20].

In this review, we will summarize the main epidemiological, pathophysiological, diagnostic, and therapeutic features of LAR, with special focus on the need for the implementation of the NAC in the clinical algorithms of rhinitis, and on the promising results of AIT as a treatment option for LAR patients. 

## 2. Epidemiology

Although large population studies are lacking, different works report that LAR is an underdiagnosed entity, affecting a considerable proportion of non-atopic rhinitis individuals of different countries, ethnic backgrounds, and age ranges [9,21,22,23,24,25,26,27,28,29,30,31,32,33,34,35]. Several studies from our group reported a prevalence of 50–75% among non-atopic individuals with nasal symptoms suggestive of allergy [9,11,23,36]. Nevertheless, the existing literature reflects an extremely wide range of LAR prevalence among non-atopic rhinitis patients (0–100%) [9,21,22,23,24,26,27,28,29,30,31,32,33,34,37]. In recent years, several studies from Asian countries have become available and overall report a lower (<20%) prevalence of LAR as compared to Western countries [26,27,29,33,37]. It has been suggested that LAR prevalence in the Mediterranean areas (Portugal, Spain, Italy, Greece, etc.) might be higher than in Northern European countries [38], yet the reported prevalence in Poland is similar to those in the Mediterranean countries [39]. Nevertheless, other factors such as diagnostic methodology or the baseline features of the patients included might also account for these discrepancies. Importantly, some studies include any non-atopic patient with rhinitis [27,29,33], whereas other works only focus on individuals who report nasal symptoms suggestive of allergy [9,23,34]. Moreover, in some studies, LAR diagnosis is based on the detection of nasal sIgE [34], whose sensitivity is considerably lower than that of the NAC. Recently, two systematic reviews and meta-analysis from Hamizan et al. have shed light into LAR prevalence. The first one including data from 3400 patients and healthy controls reports a 24.7% probability of a positive NAC in rhinitis patients testing negative for both SPT and serum sIgE [40]. The second analysis included 648 non-atopic rhinitis patients and reported a 10.2% proportion of detectable nasal sIgE among them, whereas among those with a history suggestive of allergy the proportion increased to 19.8% [41].

Fewer studies have examined the prevalence of LAR in children, yet the range frequency seems similar to that observed in adults (0–67%), with higher prevalence in the Mediterranean areas and lower prevalence in Asian countries [31,42,43,44,45,46]. A recent study from Tsilochristou et al. reports a 29.2% prevalence of LAR among a highly selected population of Greek non-atopic children with difficult-to-treat rhinitis [47]. Only one Polish study has specifically investigated the elderly population with a reported prevalence of 21% for LAR among all rhinitis patients [28]. 

Similar to AR, house dust mites (HDM) (especially *Dermatophagoides pteronyssinus*, DP) are the most common triggers of perennial LAR [35]. Grass pollen is frequently involved in the seasonal cases of LAR [9,23,36]. Interestingly, *Alternaria alternata* is most commonly involved in LAR than in AR, whereas animal epithelia and the *Olea europaea* pollen are less frequently associated with LAR as compared to AR, at least in the Mediterranean areas [9,23,36]. Similar to AR patients, nasal reactivity to several allergens can be present in LAR individuals [9,36,48]. 

## 3. Pathophysiology

The positivity of the NAC in non-atopic rhinitis patients with detectable nasal sIgE was first described by Huggins & Brostoff in 1975 [49]. In 2001–2002, this phenotype was revisited by an Australian group who also reported a similarly increased infiltrate of IgE+ cells in the nasal mucosa of both atopic and non-atopic rhinitis patients [21,50]. Studies from our group show that LAR individuals display a nasal eosinophilic inflammation and upon allergen exposure there is rapid increase and decrease of tryptase in the nasal secretions, whereas eosinophil cationic protein (ECP) increases progressively during the subsequent 24 h [9,11]. Despite the above-mentioned evidences, the involvement of sIgE in LAR pathophysiology has been questioned [51], especially due to the relatively low proportion of LAR patients with detectable nasal sIgE. On the other hand, the pooled analysis of LAR individuals demonstrated a significant increase of sIgE in the nasal secretions during the 24 h following a NAC, yet the concentration was overall very low and not all patients tested positive at least at one time point [9].

In AR patients, the allergen exposure induces a mucosal synthesis of sIgE through the sequential class switch recombination of sIgG+ memory B cells [52,53]. The locally-produced sIgE binds to the high-affinity receptor (FcεRI) expressed on resident effector cells (“sensitization”) and subsequently reaches the blood stream through the lymphatic vessels [52,54]. In the blood stream sIgE sensitizes first circulating basophils and thereafter is distributed throughout the organism to bind to FcεRI expressed on resident cells, including skin mast cells [55]. After saturating the whole receptor system, free sIgE can be found in the sera of AR individuals [56]. Therefore, serum sIgE in AR patients derives mainly from the nasal mucosa rather than from the secondary lymphoid tissue [56].

Even though these immunological phenomena have to date not been demonstrated in local allergy, there are several indirect evidences suggesting an IgE-mediated mechanisms for LAR. A proportion of LAR patients display positive basophil activation test (BAT) responses [57,58], and the addition of wortmanin (a PI3K blocker preventing IgE-dependent activation) to the test inhibits the basophil activation [59]. Moreover, and similar to AR, the majority of LAR patients respond satisfactorily to AIT [18,19,20]. On the other hand, the relatively low detection rate of nasal sIgE in LAR individuals is not surprising, as these patients test by definition negative for serum sIgE, and both biological fluids are connected through the lymphatic vessels [60]. 

**Figure 1:**
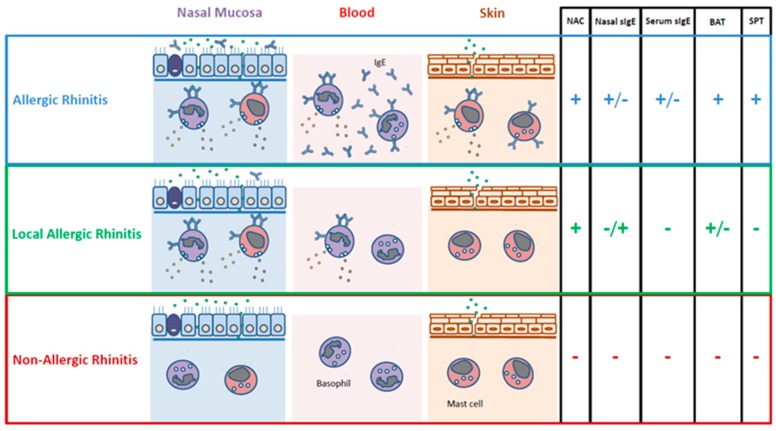
In vivo and in vitro biomarkers for rhinitis phenotypes. NAC: nasal allergen challenge; sIgE: allergen-specific IgE; BAT: basophil activation test; SPT: skin prick test.

In summary, further studies are warranted to elucidate the role of sIgE in LAR (Figure 1). The functions of other cells within the lymphoid lineage, such as innate lymphoid cells (ILC), invariant natural killer T cells, or tissue-resident memory T cells have not been investigated to date in LAR. Type 2 ILC have been related to eosinophilic nasal inflammation in humans [61], and they might have a role in reactivating memory T and B cells in LAR individuals. 

## 4. Natural Evolution and Comorbidities

LAR is a differentiated rhinitis phenotype not evolving to systemic atopy over time. A large 10-year follow-up study from our group demonstrates that the conversion rate to systemic atopy is comparable between LAR patients and the general population (9.7% vs. 7.8%, *p* = 0.623) [62,63]. Therefore, LAR is not the initial state of AR. 

LAR often starts during childhood, persists during adulthood and progresses towards the clinical worsening and the association of comorbidities in other mucosal organs [62]. The same follow-up study shows that during the first 10 years of disease evolution persistent cases of rhinitis progress from 64.8% to 88.6%, and severe cases from 18.8% to 42.0% (*p* < 0.001) [63]. Patient-reported clinical evolution and health perception worsened during the study period, and the impairment of quality of life increased from 55.1% to 85.2% (*p* < 0.001) [63]. Importantly, the allergen concentration tolerated in the NAC significantly decreased for all allergens examined [63]. At the moment of disease onset, 18.8% of patients reported symptoms suggestive of asthma, a proportion increasing to 30.7% after 10 years (*p* = 0.009) [63]. On the other hand, the cases of conjunctivitis progressed from 52.3% to 61.9% during the same period [63]. The proportion of patients requiring emergency assistance due to their nasal, ocular or bronchial disease increased from 17.6% to 42.6% (*p* < 0.001) and FEV1 decreased from 94.1% to 89.1% (*p* = 0.001) [63]. Of note, the clinical worsening occurred quicker during the first 5 years of disease evolution [62], with slower progression during the subsequent 5 years [63]. 

A recent study from our group investigated the nature of the bronchial symptoms in LAR individuals [64]. Asthma was confirmed (positive methacholine test) in 50% of LAR patients self-reporting bronchial symptoms, whereas this proportion increased to 83.3% and 57.9% in AR and NAR individuals, respectively (*p* = 0.022 AR vs. LAR) [64]. On the other hand, 28.8% and 83.3% of LAR and AR patients respectively experienced a positive response in the bronchial allergen challenge (BAC), in contrast to none of the NAR or healthy control subjects [64]. In the methacholine test performed 24 h after the BAC, there was a significant decrease in the PC_20_ as compared to the first methacholine test in all BAC+ patients (*p* = 0.016 for LAR, *p* < 0.001 for AR) but in none of the BAC- individuals [64]. This finding demonstrates the specificity of the bronchial response in BAC+ patients regardless of their atopic status. The same study also investigated the immunological features of the bronchial inflammation. The allergen administration induced a significant increase of sputum eosinophils, monocytes and ECP in BAC+ patients regardless of their atopic status, with no changes in BAC-individuals [64]. Of note, this infiltrate closely resembles that of airway allergy [65,66]. Conversely, no sIgE was detectable in the sputum of any of the study subjects [64]. Overall, these data support the existence of a bronchial counterpart of LAR (local allergic asthma) in some non-atopic asthma patients. Moreover, these findings reinforce the united airways concept [2] by demonstrating important pathophysiological links between the upper and the lower airways, also in the case of local allergy. 

A recent Japanese study suggests the existence of an ocular counterpart of LAR (local allergic conjunctivitis) in non-atopic patients with conjunctivitis and detectable total IgE in tears [67]. Nevertheless, in this study, the specificity of IgE in tears was not investigated, and conjunctival allergen challenges were not performed [67]. Moreover, the links with the nasal disease were not examined [67]. Yet epidemiological data shows that LAR patients often suffer from conjunctivitis [63], the nature of their ocular symptoms and their relationship with the allergen exposure and the nasal disease remains to be investigated.

## 5. Diagnosis

The NAC is the gold standard for LAR diagnosis, as it displays the optimal sensitivity and specificity [10,13,14]. A recent study from our group including data from 11499 procedures performed in 518 children and 5830 adults (1547 of them with asthma symptoms), demonstrated that the NAC is an extremely safe technique (99.97% of the procedures were well tolerated) [68]. Of note, the allergen administration by nasal spray or micropipette were equally safe [68]. A recent position paper of the European Academy of Allergy and Clinical Immunology has progressed in the harmonization of the NAC procedure [8]. Standardized allergen extracts should be used, and the allergen should be applied bilaterally [8,69]. The measurement of the NAC outcome should be based on both subjective (nasal-ocular symptoms) and objective (nasal patency) parameters [8,69]. To assess the nasal patency, several methods are accepted: nasal peak inspiratory flow, active anterior rhinomanometry, acoustic rhinometry, and four-phase rhinomanometry. A NAC is considered positive if the patient experiences a very significant change in symptom score or in nasal patency. The NAC can be also considered positive if moderate changes occur simultaneously in both parameters [8]. In the above-mentioned study, we also report a very high reproducibility for the NAC (97.32%, PPV 100%, NPV 92.92%) when assessed by Lebel symptoms score and acoustic rhinometry [68]. This analysis was based on three consecutive NACs performed with the same allergen in 710 patients with 1–2-month interval, to avoid the evolution bias. 

On the other hand, the NAC is a time-consuming procedure requiring technical resources and trained personnel [8]. To facilitate the implementation of the NAC in the clinical practice, our group described a protocol to perform a nasal provocation with multiple allergens in the same session (NAC-M) [48]. Importantly, when the same patient was subjected to either a NAC-M or to consecutive NACs with one allergen/session, the results of the NAC-M were 100% concordant with those of the NACs performed with single allergens (NAC-S). This finding demonstrates that the NAC-M protocol does not induce false positive results or irritant effects [48]. Interestingly, the NAC-M was associated with a 75% and 55% reduction in the number of sessions required to reach the diagnosis of NAR and LAR, respectively [48]. Of note, NAC-S and NAC-M are equally safe protocols [68].

The sensitivity of the measurement of nasal sIgE for LAR diagnosis is considerably lower than that of NAC (positive in 20–43% of LAR cases) [70,71], even when measured after allergen provocation [9,11]. Yet this low sensitivity might be partially explained by technical or dilution effects [71], it cannot be excluded that a proportion of LAR patients do not have sIgE in the nasal secretions, as previously mentioned. Of note, the published literature shows a consistent proportion of 20–25% of SPT + rhinitis patients who do not have detectable nasal sIgE (some of them even no serum sIgE) [41,72,73,74]. Several samples have been used to measure nasal sIgE (secretions, scraping, brushing, tissue homogenates, etc.) [71], yet not all of them have been applied to LAR. Therefore, nasal sIgE should be regarded mostly as a research tool, which cannot be recommended for routine LAR diagnosis [75,76,77]. 

A different diagnostic method whose performance has been investigated in LAR is the BAT. Studies from different groups report that 50–53.3% of HDM-LAR patients have positive BAT responses [58,59]. Importantly, wortmanin experiments confirmed the IgE-dependent activation of basophils [59]. Our group also reported a 66.6% sensitivity of BAT for the diagnosis *Olea europaea*-LAR patients [57]. Unlike nasal sIgE, a previous NAC is not needed to increase the sensitivity of the BAT (unpublished data), which facilitates its clinical implementation. Nevertheless, the BAT should be also considered as a research tool, and more studies are needed to validate its diagnostic performance and to assess its cost-effectiveness.

**Figure 2:**
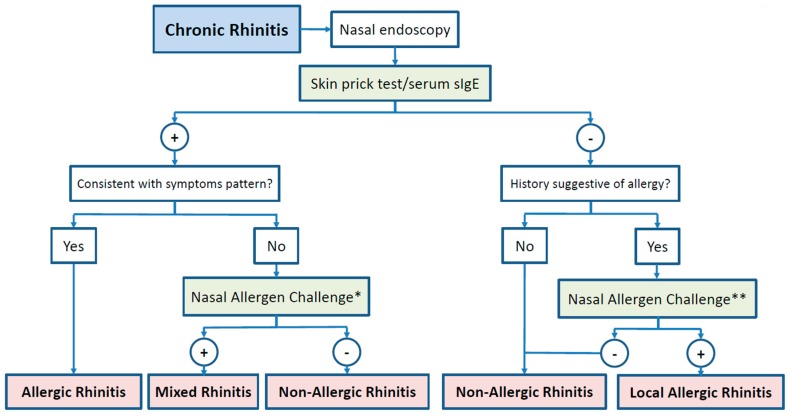
Diagnostic algorithm for chronic rhinitis. *: with the allergens positive in SPT/serum sIgE; **: with the suggestive allergens.

In summary, the NAC is the basis for LAR diagnosis, whereas nasal sIgE and BAT should be regarded mostly as research tools (Figure 2).

## 6. Treatment

No study has evaluated to date the therapeutic performance of oral antihistamines or nasal steroids in LAR patients. Nevertheless, a recent cluster analysis of rhinitis endotypes found an association between LAR and histamine metabolites [78]. Moreover, the clinical experience indicates that these drugs are similarly effective in LAR patients as compared to AR individuals. This fact is not surprising, as both entities share many clinical and pathophysiological features, including the eosinophilic nasal inflammation and the reactivity to the allergens [10]. These similarities prompted investigators to question whether AIT has a similar beneficial effect in LAR, as demonstrated for AR [17]. Four studies [18,19,20,79], including three randomized double-blind placebo-controlled clinical trials [19,20,79], have been published to date using different allergen extracts (HDM, grass, birch) (Table 1). Importantly, all studies of AIT in LAR patients have used commercially-available standardized allergen extracts. In a first open observational study from our group, 6 months of pre-seasonal subcutaneous grass pollen-immunotherapy (IT) were associated with lower symptom and medication scores, more medication free days and higher allergen concentration tolerated in the NAC during the 6 months following grass pollen-IT discontinuation [18]. At the end of the study, only the treated group had significantly higher serum grass-sIgG as compared with baseline [18].

The first controlled trial included 36 LAR patients from Spain who were randomized to receive either 2 years of subcutaneous DP-IT or placebo [19]. From the sixth month, the actively treated group displayed significantly lower symptom score, medication score, and combined symptom and medication scores (CSMS), together with higher number of medication free days [19]. Moreover, there were significant differences in the amount of allergens tolerated in the NAC from the 6th month, and at the end of the study the active group tolerated a concentration of Der p 1 >3 times higher than the placebo group [19]. The active group showed progressively increasing concentration of DP-sIgG_4_ until the end of the study period, with significant differences from the 12th month [19]. A different controlled trial included 29 Polish patients who were randomized to receive either 2 years of subcutaneous *Betula verrucosa*-IT or placebo [79]. At the end of the study period, the active group had significantly lower CSMS and higher serum Bet v 1-sIgG_4_ [79]. Interestingly, the seasonal increase of nasal sIgE at the end of the study was blunted in the active group, but not in the placebo group [79]. It might be interesting to explore the potential of this finding as a response biomarker for AIT in LAR patients [16,80].

The other controlled trial included 56 LAR individuals from 2 different centers in Spain [20]. The patients were randomized to receive either 6 months of subcutaneous *Phleum pratense*-IT (group A) or placebo (group B) followed by 6 months of wash-up period including the grass pollen season 1 (GPS1) [20]. During the second year of the study both groups received 12 months of *Phleum pratense*-IT. These 12 months included the second grass pollen season (GPS2) [20]. During the GPS1, group A had significantly lower CSMS than group B, whereas during the GPS2 both study groups had similar CSMS [20]. In the intra-group comparison, group A continued lowering the CSMS during the GPS2, yet the differences were much more pronounced for group B [20]. In this study, the *Phleum pratense*-IT reduced both the nasal and conjunctival symptoms and increased the medication free days in both study groups [20]. During the first year, only group A tolerated significantly more allergen in the NAC, whereas during the subsequent 12 months there was a progressive and parallel increase of the allergen concentration tolerated by both groups [20]. During the GPS1, there was a clinically relevant improvement of quality of life in group A, whereas group B experienced a significant worsening [20]. Conversely, both study groups reported improvements in quality of life during GPS2 [20]. Serum *Phleum pratense*- and Phl p 1-5-sIgG_4_ became progressively higher during the second year in both study groups, yet the increase was more pronounced in group A [20].

Overall, these studies suggest the ability of AIT to control LAR symptoms while the therapy is being administered. Moreover, AIT is a safe treatment option for LAR patients, as only few moderate-to-mild local reactions occurred with the administration of both active and placebo doses [18,19,20,79] Nevertheless, more studies with larger sample sizes are warranted to confirm these results. 

## 7. Conclusions

LAR constitutes a diagnostic and therapeutic challenge for clinicians. Despite affecting a significant proportion of non-atopic rhinitis patients [40], the condition remains largely unrecognized and subsequently misdiagnosed. LAR often starts during childhood and quickly progresses towards the clinical worsening and the association of comorbidities [63], most importantly local allergic asthma [64]. The first years after disease onset might constitute a window of opportunity to implement specific measures aiming to prevent disease progression and the association of comorbidities. 

LAR diagnosis relies on the positivity of the NAC [10], whereas the BAT and nasal sIgE can only assist the diagnosis in very selected cases [57]. Thus, the implementation of the NAC in the diagnostic algorithms [68] for rhinitis is a prerequisite for an early recognition of the condition and the prescription of specific therapies with disease-modifying potential. In this regard, AIT is the only existing etiological treatment for airway allergy [17], and it also seems to have the ability to control LAR symptoms while is being administered [19,20,79]. Further studies are warranted to elucidate the long-term effects of AIT on the local forms of airway allergy after therapy discontinuation, and its capacity to prevent and control asthma symptoms in LAR patients. 

## Figures and Tables

**Table 1:** Summary of the major findings of the studies investigating the effect of allergen immunotherapy in local allergic rhinitis; RDBPC: randomized, double-blind, placebo controlled; CSMS: combined symptom and medication score; sIgG_4_/IgG/IgE: allergen-specific IgG_4_/IgG/IgE; STU, DPP and AUM are standardization units used by ALK, Leti and Hal Allergy, respectively.

	Design	Comparator	Allergen Immunotherapy Extract	Allergen Dose	Primary Outcome	Rhinoconjunctivitis Symptom Score	Conjunctival Symptom Score	Medication Free Days	Quality of Life	Allergen Tolerated in the Nasal Challenge	Serun sIgG4	Nasal sIgE
Rondon, *J Allergy Clin Immunol* 2011 [18]	Open Observational	Medication only	Pangramin Plus © Grass pollen mix ALK	8 mL/month (1000 STU/mL)	Nasal tolerance to the allergen AND serum sIgG	Decrease	Not measured	Increase	Not measured	Increase	Increase (sIgG)	Not measured
Rondon, *Allergy* 2016 [19]	RDBPC	Placebo	Pangramin Plus © Dermatophagoides pteronyssinus ALK	8 mL/month (1000 STU/mL)	CSMS, symptom score, medication score, medication free days	Decrease	Not measured	Increase	Not measured	Increase	Increase	Not measured
Rondon, *Allergy* 2018 [20]	RDBPC	Placebo	Depigoid © Phleum pretense Laborat. Leti SL	5 mL/month (1000 DPP/mL)	CSMS	Decrease	Decrease	Increase	Improvement	Increase	Increase	Not measured
Bozek, *Ann Allergy Asthma Immunol* 2018 [79]	RDBPC	Placebo	Purethal© Betula verrucosa HAL Allergy SLU	5 mL/month (20000 AUM/mL)	CSMS	Decrease	Not measured	Increase	Improvement	Not measured	Increase	Decrease
